# Alteration of the Retinoid Acid-CBP Signaling Pathway in Neural Crest Induction Contributes to Enteric Nervous System Disorder

**DOI:** 10.3389/fped.2018.00382

**Published:** 2018-12-03

**Authors:** Cheng Li, Rong Hu, Nali Hou, Yi Wang, Zhili Wang, Ting Yang, Yan Gu, Mulan He, Yu Shi, Jie Chen, Weihong Song, Tingyu Li

**Affiliations:** ^1^Children's Nutrition Research Center, Children's Hospital of Chongqing Medical University, Chongqing, China; ^2^Ministry of Education Key Laboratory of Child Development and Disorders and Chongqing Key Laboratory of Translational Medical Research in Cognitive Development and Learning and Memory Disorders, Children's Hospital of Chongqing Medical University, Chongqing, China; ^3^Department of Gastrointestinal Surgery and Neonatal Surgery, Children's Hospital of Chongqing Medical University, Chongqing, China; ^4^Clinical Laboratory, Children's Hospital of Chongqing Medical University, Chongqing, China; ^5^Townsend Family Laboratories, Department of Psychiatry, University of British Columbia, Vancouver, BC, Canada

**Keywords:** retinoid acid signal, Sox10, CBP, neural crest, Hirschsprung disease

## Abstract

Hirschsprung Disease (HSCR) and/or hypoganglionosis are common pediatric disorders that arise from developmental deficiencies of enteric neural crest cells (ENCCs). Retinoid acid (RA) signaling has been shown to affect neural crest (NC) development. However, the mechanisms underlying RA deficiency-induced HSCR or hypoganglionosis are not well-defined. In this report, we found that in HSCR patient bowels, the RA nuclear receptor RARα and its interacting coregulator CREB-binding protein (CBP) were expressed in enteric neural plexuses in the normal ganglionic segment. However, the expression of these two genes was significantly inhibited in the pathological aganglionic segment. In a *Xenopus laevis* animal model, endogenous RARα interacted with CBP and was expressed in NC territory. Morpholino-mediated knockdown of RARα blocked expression of the NC marker genes Sox10 and FoxD3 and inhibited NC induction. The morphant embryos exhibited reduced nervous cells in the gastrointestinal anlage, a typical enteric nervous deficiency-associated phenotype. Injection of CBP mRNA rescued NC induction and reduced enteric nervous deficiency-associated phenotypes. Our work demonstrates that RARα regulates Sox10 expression via CBP during NC induction, and alteration of the RA-CBP signaling pathway may contribute to the development of enteric nervous system disorders.

## Introduction

Hirschsprung disease (HSCR) and/or hypoganglionosis are common congenital disorders of the enteric nervous system (1). The predominant feature of HSCR is a lack of enteric neurons in the end of the bowel; whereas hypoganglionosis refers to reduced enteric neurons throughout the entire bowel (1). Both conditions are due to developmental abnormities of the neural crest (NC) (2). The absence of nerve bodies that regulate the activity of the colon makes the affected intestinal segment unable to relax and pass stool, resulting in an obstruction. The neural crest (NC) is a group of transient pluripotent cells induced by complex morphogen signals at the neural-epidermis border (3). Border region genes such as *Pax3, Zic1, Msx1*, and *Sox10* synergistically activate downstream specifier genes and accomplish NC induction (3–5). After induction, which is usually marked by *FoxD3* or *Slug* expression, NC cells migrate out from the dorsal neural tube to different destinations where they develop into various tissues and organs such as pigment cells, cranial facial cartilage, heart outflow tract, and the enteric nervous system (5, 6).

*Xenopus laevis* is a versatile model for studying NC development because of its large size (embryo diameter 1 mm), numerous embryos in each oviposition and easy feeding (7–9). The developmental processes and molecular mechanisms of NC appear to be similar between species (10–12). In *Xenopus laevis*, NC induction initiates during late gastrulation (stage 12 according to the Nieuwkoop developmental schedule). After neural tube closure, NC cells leave the neural tube in a rostral-caudal wave (12). The NC cells in *Xenopus laevis* can either invade the gut during early phase (Stage 25–33 straight gut) or later phases (stage 40–41, at the onset of coiling) (13). Similar to other species, the enteric neuron precursor cells in *Xenopus laevis* are mainly derived from the vagal region, with minor contributions from the sacral level. These enteric neural crest cells (ENCCs) migrate into the primitive gut following the ventromedial pathway (between somites and the neural tube/notochord) (13, 14).

Retinoid acid (RA) influences various physical and pathological processes by activating the retinoic acid nuclear receptors (RARs/RXRs) (15). This heterodimer hormone receptor recruits histone acetyltransferase (HAT) or histone deacetylase (HDAC) and thereby activates or represses gene expression, respectively (16–19). CREB-binding protein (CBP) is a histone acetyltransferase (HAT) that associates with and acetylates transcriptional regulators and chromatin (20). CBP works as a coregulator of RAR hormone receptors (21–23).

The RA signaling pathway has long been known to regulate gastrointestinal nervous system development (24). In mouse models, targeted inactivation of RALDH2, a key enzyme responsible for RA synthesis, disrupts enteric nervous system development (25). Migrating NC cells express RARα which binds the RA ligand secreted by the paraxial mesoderm (26). This interactions triggers Ret (27), a key component necessary for enteric NC survival, migration and colonization (28–33). Normal Ret expression requires Sox10 binding at its upstream promoter region (34). Mutations in Sox10 have been reported to affect HSCR development (34). Whether and when RA signaling regulates Sox10 in enteric nervous system pathogenesis are not yet known. In this study, we found that RARα regulates Sox10 expression via CBP during NC induction, and alteration of the RA-CBP signaling pathway may contribute to the development enteric nervous system disorders.

## Materials and Methods

### Tissue and Patients

This study was carried out in accordance with the recommendations of the Ethics Committee of Children's Hospital of Chongqing Medical University. The protocol was approved by the Ethics Committee of Children's Hospital of Chongqing Medical University. All subjects gave written informed consent in accordance with the Declaration of Helsinki. Colon tissue from the normal and spastic segments of patients was obtained after operation on 7 boys and 2 girls that were between 2 months to 4 years old. They had no family history of the disease. Two of the cases were the short-segment type. Tissue samples were fixed with 4% paraformaldehyde and then imbedded in paraffin for immunofluorescence.

### Immunofluorescent Staining

Colon samples were sectioned at a thickness of 4 μm and pretreated with citrate buffer solution (pH 6.0) for 30 min at 95°C. After blocking in 5% BSA for 30 min, sections were incubated with primary antibodies (goat anti-RARα, Abcam ab28767, UK; rabbit anti-CBP, Novus NB100-91721, USA; mouse anti-β-Tubulin III, Santa Cruz sc-80005, USA; rabbit anti-NeuN, Millipore MABN140, USA; mouse anti-NeuN, Millipore MAB377) overnight at 4°C. Sections were washed with PBS and incubated in corresponding secondary antibodies (donkey anti-goat Alexa Fluor®594, Abcam ab150129; donkey anti-rabbit Alexa Fluor®594, Abcam ab150076; donkey anti-rabbit Alexa Fluor®488, Abcam ab150073; chicken anti-mouse Alexa Fluor®488, Invitrogen 1696214, USA; goat anti-rabbit Dylight ®488 Abbkine A23220,USA; goat anti-mouse Dylight®594, Abbkine A23410) for 1.5 h at 22°C.

### RNA Extraction and Real-Time PCR

Total RNA samples were extracted from the colon of patients using the TriZol™ Total RNA Extraction kit (Ambion, USA). mRNA was reverse-transcribed into cDNA using the PrimeScript™ RT Reagent kit (TaKaRa Bio, Japan), and real-time PCR reactions were carried out using a RealMasterMix™ kit (TaKaRa Bio). Primer sequences were: Human RARα forward: 5′-tccgccgcagcatccagaagaacat, reverse: 5′-actcgggcttgggcacctccttctt; Human CBP forward: 5′-acccaggcctcctcaatagt, reverse: 5′-tggagtagggtacggcattc; and Human β-actin forward: 5′-gtgaaggtgacagcagtcggtt, reverse: 5′-gagaagtggggtggcttttagga.

### Plasmid Construction

*Xenopus laevis* RARα and CBP coding regions were obtained by PCR and cloned into the pGMT vector to prepare probes. The following primers were used: *Xenopus laevis* RARα forward 5′-cagcctattcccgtgccaand, reverse 5′-catcgtgtcggtctgtcctt; and *Xenopus laevis* CBP forward 5′-gaatccttaccctttcgtcagcc and reverse 5′-cagccggatggcaatggaag. Full-length *Xenopus laevis* RARα and CBP open reading frames were cloned into the pCS2+ expression vector for rescue experiments. The rescue plasmids do not contain the sequences targeted by RARα- and CBP-specific morpholino oligomers (MOs). The following primers were used for full length rescue: RARα: sense5′-ccggaattccagcctattcccgtgcca, reverse 5′-tgctctagacatcgtgtccgtctgtcctt; and CBP: sense 5′-atttaggtgacactatag, reverse 5′-attaaccctcactaaaggga.

### Microinjection, Rescue, and Whole-Mount *in situ* Hybridization

This study was performed in accordance with the recommendations of the Animal Experimentation Ethical Committee of Chongqing Medical University. *in vitro* fertilization and culture of embryos, whole-mount *in situ* hybridization, and microinjection were performed as described previously (35). All MOs were designed and purchased from GeneTools LLC (USA). The sequences of MOs were: RARα-MO 5′-cgtccacattctcatacatcctaaa; CBP-MO 5′- gttctcggccatcttcactcctttc; and Control-MO 5′- cctcttacctcagttacaatttata. RARα-MO (6–24 ng per cell) or CBP-MO (10 ng per cell) were injected in two-cell or four-cell stage embryos unilaterally, and full length RARα or CBP mRNA (100–300 pg) were used for rescue. LacZ was coinjected to trace the injected sides. Previously reported probes were used for *in situ* hybridization to detect the expression of Pax3, Zic1 (36), FoxD3 (37), Sox10 (38), and N-tubulin (39).

### Coimmunoprecipitation and Western Blotting

Embryos were lysed with 1% Triton-X100 in phosphate-buffered saline containing a protease inhibitor cocktail. For immunoprecipitation analysis, 600–1200 μg of protein was incubated with the anti-CBP antibody (Abcam ab50702, UK) or IgG and protein A/G plus agarose beads (Santa Cruz sc-2003, USA) overnight at 4°C. The immunoprecipitation reactions were washed five times and boiled for western blotting. Approximately 80–120 μg of protein was resolved on 10% SDS-PAGE gels and transferred to 0.45 μm PVDF membranes. All membranes were blocked in 5% marvel milk in TBST and then incubated with appropriate antibodies at 4°C overnight. Primary antibodies were used to detect RARα (Novus NBP2-45516, USA) and CBP (Novus NB100-91721).

### Statistical Analyses

All experiments were carried out at least three times. The Student's *t*-test was used, and *p* < 0.05 was considered to be statistically significant.

## Results

### Absent RARα and CBP Expression in Pathological Aganglionic Segments of HSCR Patients

**Figure 1:**
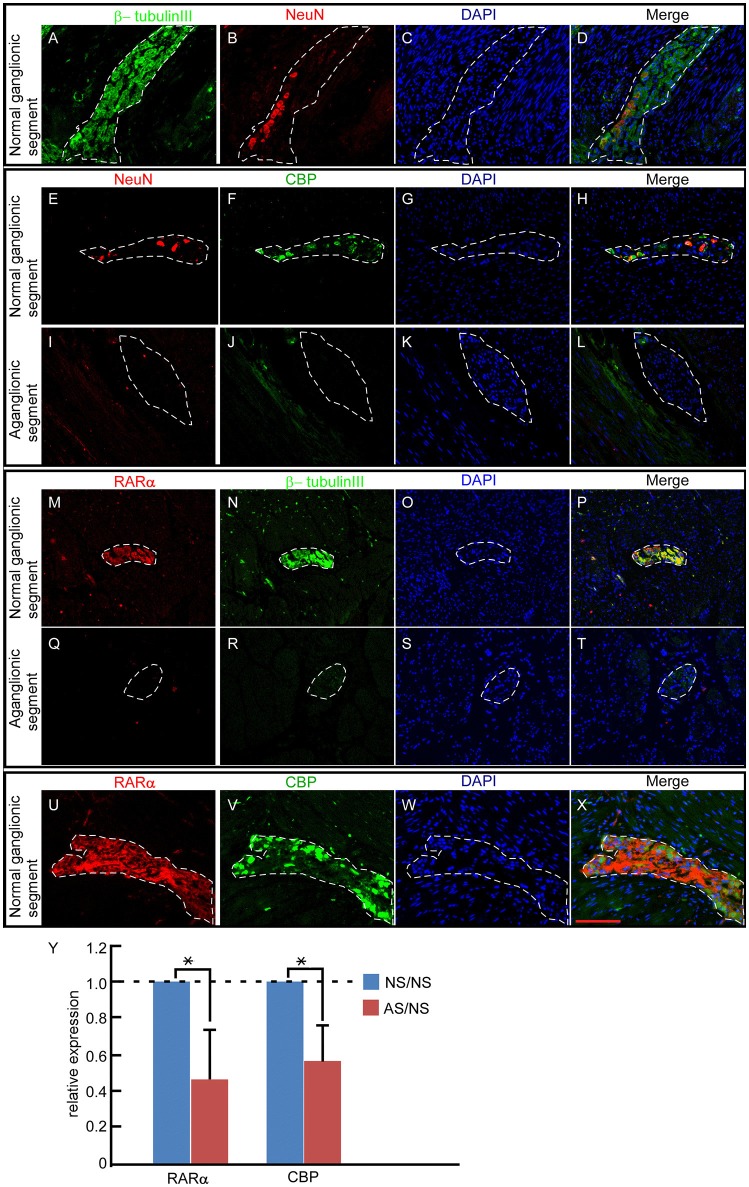
RARα and CBP expression patterns in the enteric nervous system of HSCR patients. **(A–D)** In normal ganglionic segments, β-tubulin III (green)-labeled nerve fibers **(A)** and NeuN protein (red) indicate ganglion cells **(B)**. DAPI-stained cell nuclei **(C)** β-tubulin III and NeuN displayed partially overlapping expression patterns in the neural plexus, as shown in the merged panel **(D)**. **(E–H)** In normal ganglionic segments, CBP protein (green) **(F)**-labeled ganglion cells are detected by colocalization **(H)** with NeuN (red) **(E)**. **(I–L)** In aganglionic segments, NeuN **(I)** and CBP **(J)** are absent within the neural plexus, and CBP is expressed faintly in the muscularis propria of the colon, as shown in the merged panel **(L)**. **(M–P)** In normal ganglionic segments, RARα **(M)** colocalizes with β-tubulin III **(N)**, as shown in the merged panel **(P)**. **(Q–T)** RARα **(Q)** is absent and β-tubulin III **(R)** is weakly expressed in aganglionic segments. **(U–X)** In normal ganglionic segments, RARα **(U)** labels nerve fibers and CBP **(V)** indicates ganglion cells. **(X)** Both proteins display partially overlapping patterns within the neural plexus. **(Y)** RARα (0.459 ± 0.273) and CBP (0.569 ± 0.196) transcripts were significantly lower in aganglionic segments than in normal regions. NS, normal ganglionic segments; AS, aganglionic segments. Numbers in brackets indicate the relative expression ratio of pathogenic aganglionic segments to normal ganglionic segments. Dashed lines encircle the neural plexus. ^**^P < 0.01, with *T*-test. Scale bar: 100 μm.

To explore roles of RARα and CBP in enteric nervous system development, we first investigated the enteric expression patterns in excision bowels from 9 cases HSCR in children. Each bowel contains ganglionic (normal control) and aganglionic (pathological) segments. All 9 cases exhibited similar expression patterns. In the ganglionic segments, NC-derived ganglion cells expressed clear NeuN protein (Figure 1B red), consistent with previous reports (40, 41). Cell nuclear was stained by DAPI (Figures 1C,G,K,O,S,W). Beta-tubulin III and NeuN displayed partially overlapping expression in the merged panel (Figure 1D). CBP-positive cells (Figure 1F green) overlapped perfectly with NeuN-expressing cells (Figures 1E,H), suggesting that ganglion cells expressed CBP protein. These ganglion cells were arranged along the nerve fibers as detected by β-tubulin III staining (Figure 1A green) (42, 43). In addition, RARα protein (Figure 1M) exhibited the same expression pattern as β-tubulin III (Figures 1N,P). In the aganglionic distal bowel, neither NeuN (Figure 1I, dash line) nor CBP (Figure 1J, dash line) was observed in the neural plexus (Figure 1L, dash line). CBP was weakly expressed in the muscularis propria region (Figure 1J). Previous work described decreased expression levels of β-tubulin in HSCR patients (44). In our study, expression of β-tubulin III (Figure 1R, dash line) as well as RARα (Figure 1Q, dash line) was diminished in the aganglionic segments. Similar to the protein level, the ratio of mRNA levels in pathogenic aganglinoic segments to normal ganglionic segments are 0.459 ± 0.273 for RARα (^*^P < 0.05, *n* = 9) and 0.569 ± 0.196 for CBP (^*^p < 0.05, *n* = 9) (Figure 1Y). The partially overlapping expression pattern of RARα and CBP in the normal ganglionic segment (Figures 1U–X) and their shared diminishment in the pathological aganglionic segment suggests that RARα and CBP might play a role in enteric ganglion cell development and/or function.

### Expression of RARα and CBP in the Developing NC Cells

**Figure 2:**
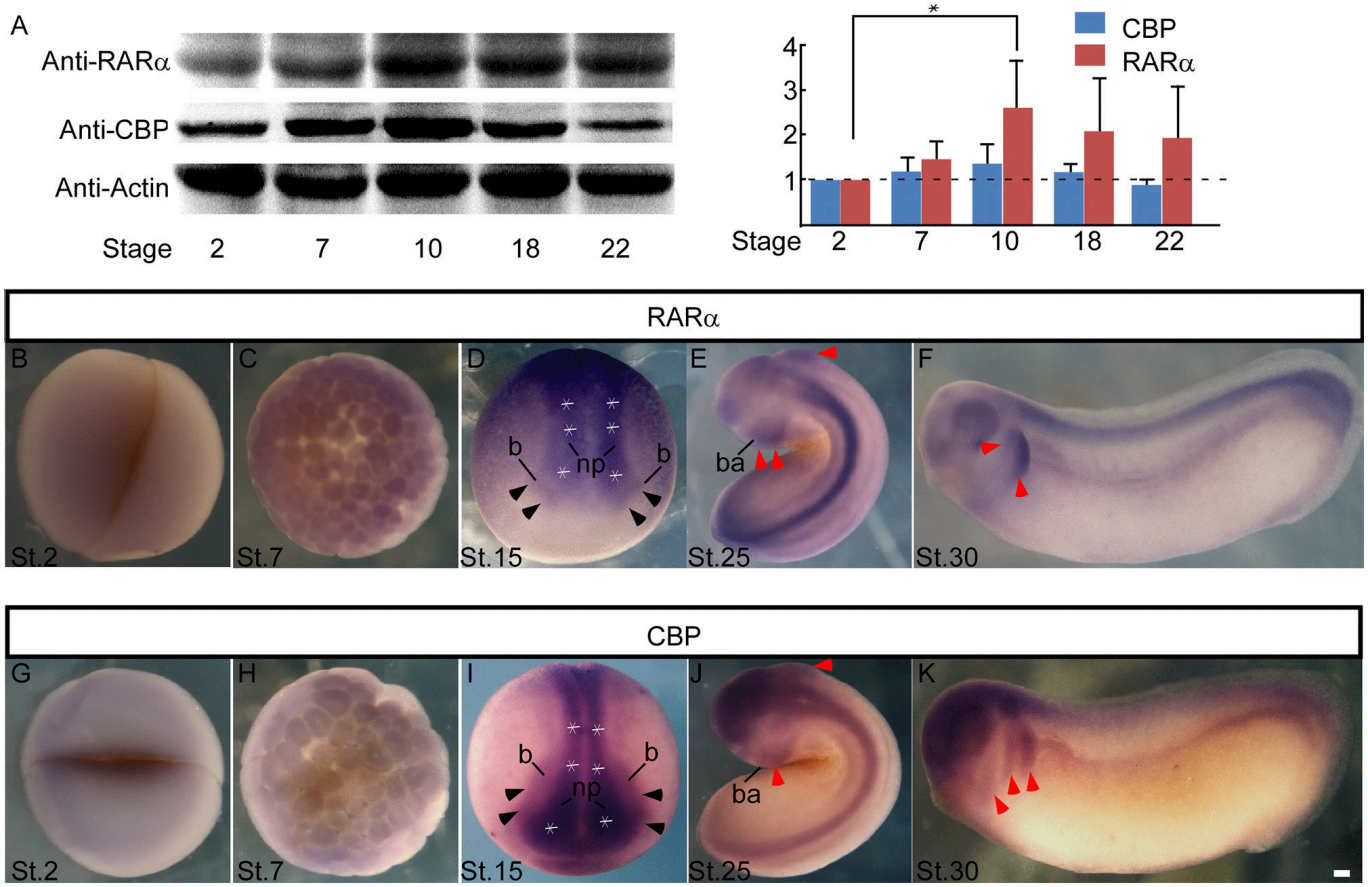
RARα and CBP expression in presumptive neural crest in *Xenopus Laevis*. **(A)** Developmental expression of the RARα and CBP proteins in *Xenopus laevis*. β-actin was used as an internal control. Statistics revealed the ratio of protein levels relative to stage 2. RARα **(B)** and CBP **(G)** are maternally expressed at the animal hemisphere, and the expression intensity increases at stage 7 **(C,H)**. At the neural plate stage (stage 15), RARα **(D)** and CBP **(I)** appear in the central neural plate (^*^ white asterisk means neural plate) and anterior neural plate border (black arrowhead). At stage 25 **(E)** and stage 30 **(F)**, RARα is in the neural tube and branchial arch (red arrowhead). CBP has a similar expression pattern in the branchial arch (red arrowhead) and neural tube at stage 25 **(J)** and stage 30 **(K)**. np, neural plate; b, neural plate border region; ba, branchial arch. Scale bar: 100 μm.

Enteric ganglion cells derive from NC cells. To explore the involvement of RARα and CBP in NC development, we first checked the expression pattern of these two genes in *Xenopus laevis*. RARα and CBP proteins shared similar temporal expression patterns during early embryonic development. Both RARα and CBP proteins were maternally expressed (relative expression level at stage 2 designated as 1, and the following numbers represent protein intensity ratios to stage 2), and the levels increased at the onset of the mid-blastula-transition (stage 7) (RARα 1.46 ± 0.39, CBP 1.17 ± 0.33) when zygotic transcription begins. Their expression peaked at the beginning of gastrulation (stage 10) (RARα 2.57 ± 1.08, CBP 1.35 ± 0.43) followed by a slight decrease at the later neurula stage (stage 22) (RARα 1.92 ± 1.16, CBP 0.88 ± 0.14P) (Figure 2A). Whole mount *in situ* hybridization assays revealed the localization of RARα and CBP in the animal hemisphere at stage 2 (Figures 2B,**G**) and stage 7 (Figures 2C,H). At the neural-plate stage, RARα and CBP were expressed in the neural plate (Figures 2D,**I**, white asterisk) and plate-epidermis border region (Figures 2D,**I**, black arrowhead) where NC is induced. After neural tube closure, the branchial arch, which is composed of migrating NC cells, expressed RARα (Figures 2E,**F**, red arrowhead) and CBP (Figures 2J,**K**, red arrowhead). Our data suggest that RARα and CBP are expressed in the developing NC cells.

### RARα and CBP Are Required for Enteric Nervous Cell Development

**Figure 3:**
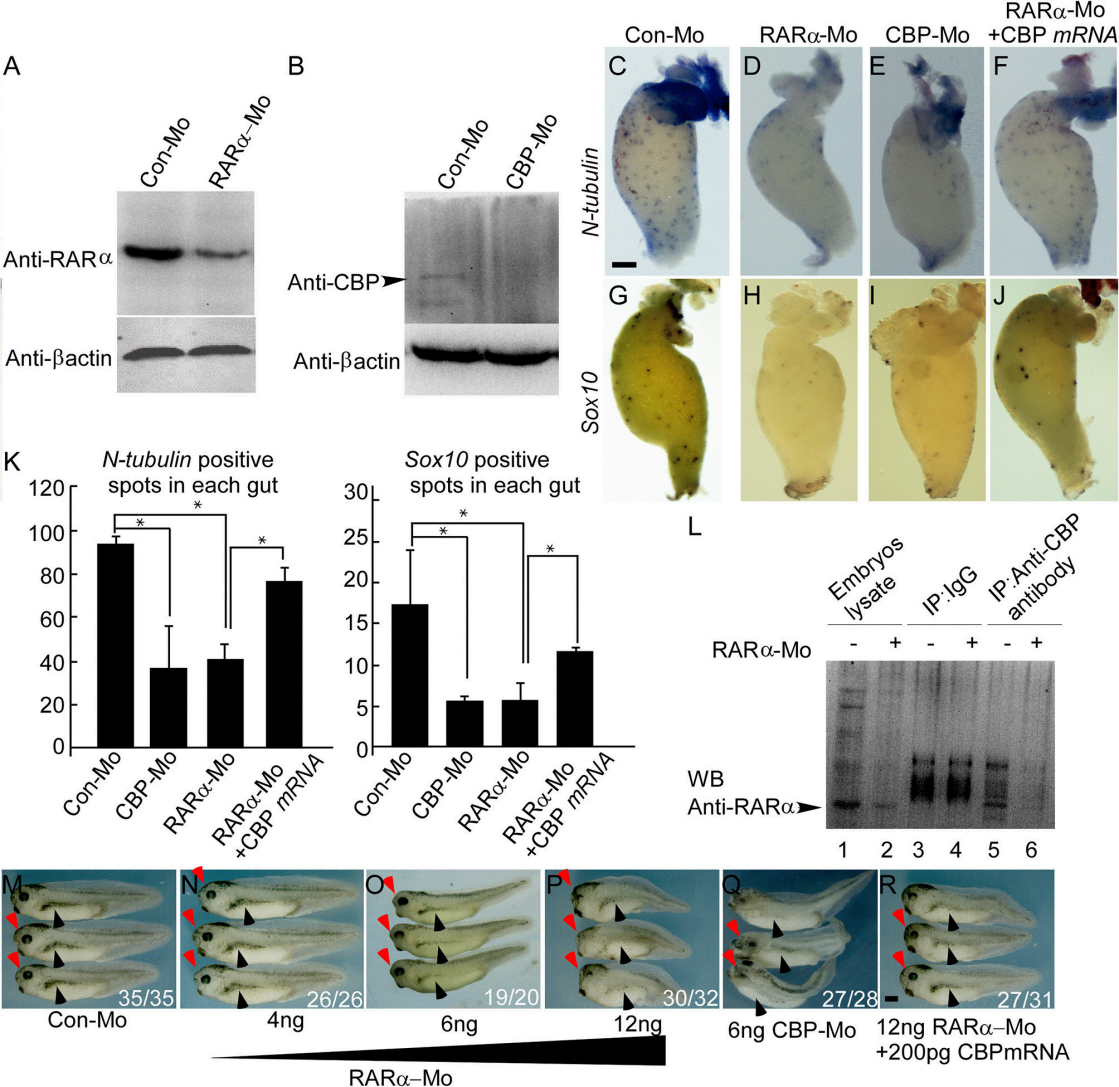
RARα and CBP are cooperatively required for enteric nervous cell development in *Xenopus laevis*. Morpholino-mediated knockdown of RARα **(A)** or CBP **(B)** is efficient at embryonic stage 15. **(C–J)** RARα **(D,H)** or CBP **(E,I)** knockdown decreases enteric nervous cells (labeled with N-tubulin) and enteric neural crest cells (labeled with Sox10) compared to control guts **(C,G)**. CBP mRNA coinjection rescues RARα morpholino effects on the gut **(F,J)**. Gut anlage was harvested at stage 41. Enteric nervous cells are detected as dispersed blue-spots by N-tubulin *in situ* hybridization, whereas enteric neural crest are marked by Sox10. **(K)** Statistical analysis of enteric nervous cells among different groups. Nervous cells were quantified by counting the number of blue spots. In each group, at least three guts were analyzed and presented as the mean ± sd. **(L)** RARα coimmunoprecipitates with CBP in stage 15 *Xenopus laevis* embryos. **(M–R)** RARα morpholino injection at the 2- or 4-cell stage into the dorsal blastomere leads to dose-dependent microcephaly (red arrowhead) and pigment cell abnormalities (black arrowhead) at stage 37. **(Q)** CBP morpholino injection has similar phenotypes to RARα knockdown. **(R)** CBP mRNA largely rescues the RARα morphant small head and pigment deformity phenotypes. The numbers of embryos showing similar phenotypes and total injected embryos in each group are indicated. Scale bar: 100 μm. ^*^P < 0.05.

Our data in HSCR patients indicated that RARα and CBP proteins were absent in pathological aganglionic segments, suggesting that RARα and CBP may participate in enteric nervous system development. To provide functional evidence, we knocked down RARα and CBP translation with microinjection of specific morpholinos and examined enteric nervous system development in a *Xenopus laevis* model. There are two RARα isoforms in *Xenopus laevis*, and RARα2 is the only detectable isoform after gastrulation (45). Therefore, we designed a morpholino targeting RARα2 (designated as RARα in the following text). The RARα2- and CBP-specific morpholinos efficiently knocked down RARα expression (Figures 3A,**H**) and inhibited CBP expression (Figure 3B) at stage 15, respectively. We evaluated enteric nervous system development by calculating the colony of enteric neural precursors/NC cells (labeled with Sox10) (46) and differentiated enteric nervous cells (labeled with N-tubulin) (39) in the gut anlage (stage 41). In control embryos, the number of Sox10-labeled precursor cells was 17.3 ± 6.66 per gut (Figures 3G,K), while there were 93.6 ± 4.04 N-tubulin-positive spots in each embryo gut anlage (Figures 3C,3K). Both RARα and CBP knock-down embryos displayed greatly reduced enteric neuron precursor cells (Sox10-positive blue spots 5.33 ± 0.57/gut in CBP morphant and 5.66 ± 2.08/gut in RARα morphant) (Figures 3H,I,K). The colony numbers of differentiated nervous cells, as illustrated by N-tubulin *in situ* hybridization, were consequently decreased (positive blue spots 41.3 ± 7.23/gut in RARα morphants, Figures 3D,K and 37 ± 19.2/gut in CBP morphants Figures 3E,3K) in the developing gut anlage (*P* < 0.05), which is a hypoganglionosis-like phenotype. Coinjection of CBP mRNA significantly rescued Sox10-(11.7 ± 0.58) and N-tubulin-(77 ± 6.24) positive colony numbers in RARα morphant embryos (*P* < 0.05) (Figures 3F,J). We also observed microcephaly (Figures 3M–Q red arrowhead) and pigment cell abnormalities (Figures 3M–Q black arrowhead) in RARα and CBP knock-down embryos. The severity of RARα-MO phenotypes was dose-dependent.

Previous work in our lab identified an interaction between RARα and CBP in a rat model (47). It has long been known that the RA receptor regulates CBP expression (48). We found that *Xenopus laevis* CBP co-immunoprecipitated with RARα (Figure 3L). Microinjection of CBP mRNA rescued the RARα morphant phenotypes in the head, pigment cells, and enteric nervous system (Figures 3R,F,J).

### CBP Functions Downstream of RARα in Regulating NC Induction

**Figure 4:**
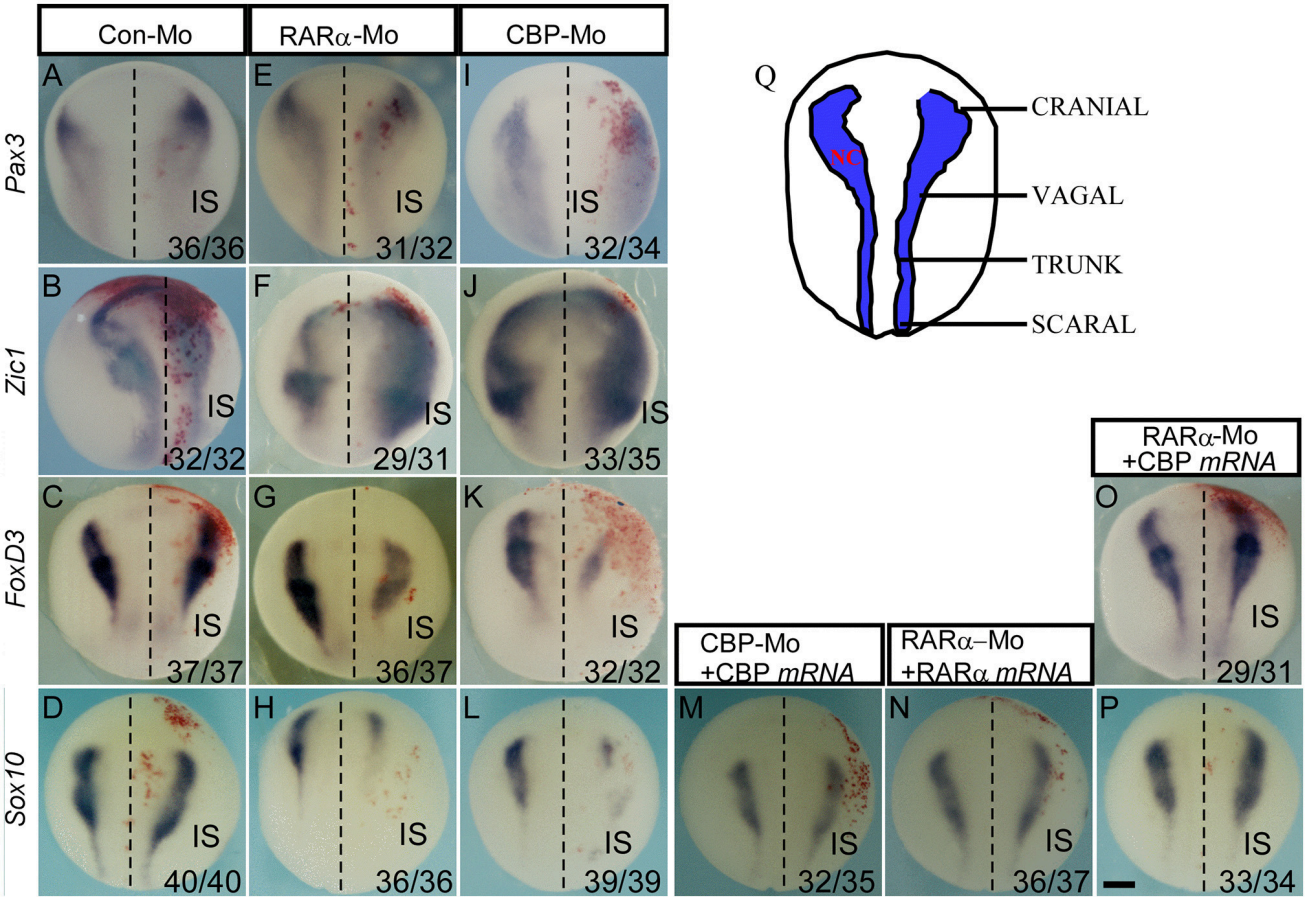
CBP functions downstream of RARα in regulating neural crest induction. The effects of the control morpholino **(A–D)**, RARα knockdown **(E–H)** and CBP knockdown **(I–L)** on the expression of indicated neural border and neural crest markers. **(M,N)** The RAR and CBP morpholino-induced reduction in Sox10 is rescued by coinjection of morpholino-resistant RAR and CBP mRNAs, respectively. **(O,P)** The RARα knockdown-induced inhibition of FoxD3 and Sox10 is rescued by CBP mRNA coinjection. All of the embryos were collected at stage 15–16. **(Q)** Schematics of neural crest from the dorsal view along the anterior-posterior axis at stage 15. IS means injected side, and the left and right embryo halves are denoted with the dashed line. The numbers of embryos showing similar changes of gene expression and the total injected embryos in each group are indicated. Scale bar: 100 μm.

We demonstrated that developing NC cells express CBP and RARα, and inhibition of CBP and RARα expression causes developmental abnormities of NC-derived enteric nervous cells, cranial facial structures, and pigment cells. We next investigated the temporal requirements and functions of RARα and CBP in NC development. At stage 15 (the NC induction stage), knockdown of either CBP or RARα did not affect Pax3 expression (Figures 4E,I) but did expand the Zic1-expression domain (Figures 4F,J). Sox10 (Figures 4H,L) and FoxD3 (Figure 4G,K) expression were inhibited in the morpholino-injected side. There was no effect of control morpholino on Pax3/ Zic1/ FoxD3/ Sox10 (Figures 4A–D). Such inhibition affects all NC regions including the cranial, vagal, trunk, and sacral areas as shown in the schematic (Figure 4Q) and subsequently affects enteric NC development. In addition, reduced expression of Sox10 and FoxD3 in RARα and CBP morphants was rescued by exogenous injection of morpholino-resistant RARα and CBP mRNAs (Figures 4M,N). NC induction is marked by FoxD3 and/or Slug expression (37, 49, 50), which lead to the expression of numerous downstream transcription factors, such as Sox10 (51). Our data suggests that knockdown of either RARα or CBP affects NC induction. CBP mRNA coinjection rescues the reduced Sox10 (Figure 4P) and FoxD3 (Figure 4O) expression patterns in RARα morphants. Such rescue indicates that CBP functions downstream of RARα during NC induction and partially accounts for rescue of the enteric nervous system development phenotypes at later stages.

## Discussion

In this work, we first demonstrated that RARα and CBP are normally expressed in the gastrointestinal ganglionic segment. In pathogenic aganglionic segments, RARα and CBP are reduced or even absent, suggesting potential functions for these proteins in development and/or maintenance of the enteric nervous system. The gastrointestinal nervous system derives from NC (52). In chick, NC cells express RARα (26). Our work in *Xenopus laevis* revealed that RARα and its interactor CBP are expressed in the NC territory. The similar expression patterns of RARα in different species suggests evolutionarily conserved roles for RA signaling in NC and enteric nervous system development.

Previous studies have showed that RA signaling affects cell polarization, lamellipodia formation and PTEN regulation, thereby affecting enteric NC migration (24). Inhibition of RA synthesis (25) or deletion of the vitamin A-binding protein (24) blocks NC migration and leads to HSCR-associated phenotypes. In this study, knockdown of either RARα or its interactor CBP leads to failure of NC induction detected with reduced Sox10 and FoxD3 expression at the border region. Both morphant embryos also presented anterior-posterior body axis deformities and reduced anterior structures, consistent with previously reported RARα morphant phenotypes (53). NC cells contribute to craniofacial cartilage genesis, which defines the scale of anterior head structures in the embryonic stage. Thus, abnormal NC induction caused by RARα or CBP morpholinos accounts for the previously reported small head phenotype. At later stages (stage 41), both morphant embryos showed decreased enteric neural precursors/ NC cells and differentiated enteric nervous cells, which is a classic HSCR-associated phenotype. Injection of CBP mRNA rescued Sox10 and FoxD3 expression at the time of NC induction time and rescued subsequent enteric nervous system defects in RARα morphants. These data clearly indicate that CBP functions downstream of RARα in regulating Sox10 expression during NC induction.

It has been shown that RA binds RARα and then activates Ret (27). Activation of Ret gene expression requires Sox10 binding at the Ret promoter region (34). Ret is an indispensable gene in enteric NC development. Our data demonstrate the mechanism by which RARα triggers Ret expression in HSCR pathogenesis. During NC induction, RARα regulates Sox10 expression *via* CBP, and subsequently Sox10 binds the Ret upstream promoter region and initiates Ret transcription.

Based on the present data, RA signaling regulates enteric NC development at two stages: it first controls NC induction and later regulates enteric NC migration. Our study indicates that vitamin A and related metabolites may be risk factor for the penetrance and expressivity of HSCR disease. Optimizing maternal nutrition levels may prevent enteric neural system disorders.

## Author Contributions

CL, YS, TL, and WS conceived and designed the experiments. CL, YS, RH, NH, YW, ZW, TY, YG, MH, and JC performed the experiments. CL, YS, JC, TL, and WS analyzed and contributed reagents, materials, or analysis tools. CL, YS, JC, TL, and WS wrote the paper. All authors reviewed the manuscript.

### Conflict of Interest Statement

The authors declare that the research was conducted in the absence of any commercial or financial relationships that could be construed as a potential conflict of interest.
